# Peritoneal Adipocytes and Their Role in Inflammation during Peritoneal Dialysis

**DOI:** 10.1155/2010/495416

**Published:** 2010-05-05

**Authors:** Kar Neng Lai, Joseph C. K. Leung

**Affiliations:** Department of Medicine, Queen Mary Hospital, University of Hong Kong, 102 Pokfulam Road, Hong Kong

## Abstract

Adipose tissue is a major site of chronic inflammation associated with peritoneal dialysis (PD) frequently complicating peritonitis. Adiposity-associated inflammation plays a significant contributory role in the development of chronic inflammation in patients undergoing maintenance PD. However, the molecular and cellular mechanisms of this link remain uncertain. Adipose tissue synthesizes different adipokines and cytokines that orchestrate and regulate inflammation, insulin action, and glucose metabolism locally and systemically. In return, inflammation retards adipocyte differentiation and further exacerbates adipose dysfunction and inflammation. An understanding of the inflammatory roles played by adipose tissue during PD and the healing mechanism of injured mesothelium will help to devise new therapeutic approach to slow the progression of peritoneal damage during peritoneal dialysis. This article reviews the roles of peritoneal adipose tissue in chronic peritoneal inflammation under PD and in serosal repair during PD.

## 1. Introduction

Continuous ambulatory peritoneal dialysis (CAPD) has emerged as a major treatment modality in renal replacement program worldwide. It has distinct advantages over hemodialysis with a lower cost and simplicity of the technique. The ability to maintain the functional integrity of the peritoneal membrane allowing effective removal of fluid and metabolic waste is essential for the success of the treatment. Unfortunately, the peritoneal membrane frequently exhibits structurally changes following long-term dialysis due to the exposure of unphysiologic peritoneal dialysis fluid (PDF) with low pH and high glucose [1]. PDF also contains toxic substances including glucose degradation products (GDP) generated during the sterilization process and advanced glycation end products (AGE) produced from Amadori reaction between sugar and protein during long-term peritoneal dialysis (PD) [2]. These compounds cause irreversible damage to the peritoneal tissue leading to ultrafiltration failure and decline in dialysis efficacy [3, 4]. Previous studies have reported the detrimental effects of PDF on peritoneal cells including human peritoneal mesothelial cells (HPMC) [5–7] and endothelial cells [8, 9]. While adipose tissue is ubiquitously present in peritoneal tissue, information for the characteristics and pathophysiology of adipocytes following long-term exposure to PDF in maintenance CAPD remains scarce. Only until recently, adipocytes are considered as passive tissue for the storage of energy in the form of fat. However, there are now compelling evidences suggesting that adipocytes exert important metabolic and proinflammatory effects on peripheral tissue [10–12]. Furthermore, peritoneal adipocytes affect HPMC through the release of adipokines and, hence, alter the peritoneal physiology during PD [13, 14].

## 2. Peritoneal Adipocytes

The parietal and visceral peritoneal surfaces are covered by a monolayer of mesothelium composed of mesothelial cells. Beneath the mesothelial cells are the basement membrane and submesothelial layer that contains collagen, fibroblasts, adipose tissue, blood vessels, and lymphatics [15]. Adipose tissue is abundant in omental or mesenteric peritoneum but less so in parietal, intestinal, and diaphragmatic peritoneum. Contrary to the prevailing view that adipose tissue functions only as an energy storage depot, compelling evidence reveals that adipocytes can mediate various physiological processes through secretion of an array of mediators and adipokines that include leptin, adiponectin, resistin, tumor necrosis factor-*α* (TNF-*α*), interleukin (IL)-6, transforming growth factor-*β* (TGF-*β*), vascular endothelial growth factor (VEGF), hepatocyte growth factor (HGF), and other growth factors [16]. Moreover, adipocytes express receptors for leptin, insulin growth factor-1 (IGF-1), TNF-*α*, IL-6, TGF-*β* and may form a network of local autocrine, paracrine, and endocrine signals [17]. All of these adipokines exert important endocrine functions in chronic kidney diseases and may also contribute to systemic inflammation in these patients. This is of special significance in patients undergoing CAPD as the initiation of treatment is often associated with an increase in fat mass that could be associated with a polymorphism in uncoupling protein 2 which affects the energy metabolism in addition to glucose absorption from the PDF [18]. In contrast to findings in the general population, a number of studies have suggested that a higher body mass index (BMI) is associated with a better outcome in patients with kidney diseases [19]. Critical analysis reveals that the protective effect from a high BMI only applies to patients with a normal or high muscle mass [20]. A recent study indicates that an increased fat mass in PD, like in other patient groups, may indeed have adverse metabolic consequences with increased systemic inflammation and worst survival [21]. Interestingly, there is a difference in the release of growth factors between visceral and subcutaneous adipose tissue [22]. The omental adipose tissue, most affected by PD, releases IL-6 two to three folds higher than the subcutaneous fat tissue [23]. The visceral (truncal) fat mass correlates significantly with circulating IL-6 levels but not for nontruncal fat mass [24]. 

Ultrastructural study reveals that a portion of omental adipocytes protrude from the mesothelial surface, thus may come into direct contact with dialysate [15]. In addition, dialysate may also reach the parietal adipose tissue when the mesothelial monolayer is damaged. It is therefore logical to postulate that with repeated exposure to PDF and the continuous change in peritoneal physiology during CAPD, peritoneal adipocytes will inevitably be “activated”. Although much work has focused on peritoneal mesothelial cells, scant attention has been paid to the role of peritoneal adipocytes during CAPD.

## 3. Stem Cells from Adipose Tissue

The stromal vascular fraction (SVF) is a heterogeneous cell population derived from the adipose tissue including omentum [25–27]. SVF is reported to be composed of endothelial cells identified as CD34+/CD31+ cells, infiltrating/resident macrophages defined as CD14+/CD31+ cells, and a population characterized as CD34+/CD31− cells. The CD34+/CD31− subset is a unique cell fraction capable of differentiating into adipocytes and is restricted to cells that do not express the mesenchymal stem cell marker CD105 [28]. It has been suggested that the adipocyte progenitor cells, that is, the preadipocytes, are included in the CD34+/CD31− cell fraction. This unique population is distinct from the multipotent adipose tissue-derived mesenchymal stem cells, which can be differentiated in vitro into other cell types including adipocytes [27], chondrocytes [29], osteoblasts [30, 31], and cardiomyocytes [32, 33]. The cellular number of SVF varies among individuals and so far there is no data studying whether PD alters the number of SVF in different adipose depots. Apart from the SVF, milky spots of the omentum also harbor stem cells [34], which proliferate to form the resident macrophage during peritoneal inflammation [35]. It remains unknown whether stem cells from milky spots have the same identity as stem cells in SVF with adipogenic potential. Milky spots are very small omental tissues in contact with peritoneal membrane, consisting of macrophages, lymphocytes, and plasma cells supported by blood and lymphatic vessels. Milky spots play a role in peritoneal infection and abdominal tumors [36, 37]. PD also activates the milky spots resulting in an increase in number and size during inflammatory process and PD [37, 38]. Milky spots transform into a lymph node-like structure where lymphocytes constitute the main cellular component after an episode of peritonitis [34].

## 4. Crosstalk between Peritoneal Cells and Adipocytes with a Focus on Leptin

Adipose tissues express and secrete a variety of cytokines and adipokines, which act locally as autocrine/paracrine mediators or systemically as endocrine factors (Table 1). Patients on PD have increased fat mass due to glucose absorption from the PDF. Increase in adiposity has been associated with sub-clinical inflammation with elevated adipokines synthesis. Among these adipokines, leptin is of particular interest as this peptide hormone is most abundant adipokine produced by adipocytes and is cleared principally by the kidney. The serum leptin concentration is increased in patients with chronic renal failure or undergoing dialysis [39, 40] and the serum leptin increases by 189% within a month after the initiation of PD treatment [41]. Leptin is also elevated during acute infection, in response to proinflammatory cytokines including IL-1 and TNF-*α* [39]. In the kidney, leptin stimulates cell proliferation and synthesis of collagen IV and TGF-*β* in glomerular endothelial cells. In glomerular mesangial cells, leptin increases the glucose transport, up-regulates the expression of the TGF-*β* type II receptor and the synthesis of collagen I through phosphatidylinositol-3-kinase related pathway [39]. Available data suggests that leptin triggers a paracrine interaction between glomerular endothelial and mesangial cells through the increased synthesis of TGF-*β* in glomerular endothelial cells and up-regulated TGF-*β* receptor expression in mesangial cells. It remains unclear whether such paracrine interaction operates between peritoneal adipocytes and HPMC. To the best of our knowledge, there is only one previous study on the effect of PDF on adipocytes that demonstrates increased leptin synthesis in a murine adipocyte cell line (3T3-L1) by glucose-containing PDF [42]. It is likely that pro-inflammatory mediators released by HPMC upon exposure to PDF could induce functional alteration of adjacent adipocytes. The likely candidates are IL-1 and TNF-*α*, TGF-*β*, VEGF, and IL-6. Indeed, a recent in vitro study has shown that IL-6 modulates leptin production and lipid metabolism in human adipose tissue [43]. Using cultured HPMC and SVF, we have shown that high glucose content in dialysate fluid is one of the major culprits that causes structural and functional abnormalities in peritoneal cells during CAPD [13, 44, 45]. Glucose significantly increases the protein synthesis of leptin by adipocytes in a dose-dependent manner and up-regulates the expression of leptin receptor, Ob-Rb, in HPMC [13]. The increased leptin production by adipocytes and enhanced Ob-Rb expression in HPMC following exposure to glucose suggest the existence of a cross-talk mechanism between adipocytes and mesothelial cells that may be relevant in peritoneal membrane dysfunction developed during peritoneal dialysis. HPMC cultured with conventional PDF induce higher expression of VEGF than that experiments with low-GDP-content PDF. In parallel, GDPs increase the gene and/or protein expression of VEGF in HPMC [46]. GDPs also decrease the expression of proteins associated with the tight junction, zonula occludens protein 1 (ZO-1), in HPMC [44]. Exogenous VEGF down-regulates the expression of ZO-1 while neutralizing anti-VEGF antibody reverses the effect of GDPs on ZO-1 expression in HPMC. These findings suggest that the action of GDPs on ZO-1 expression is mediated through VEGF. 

A longitudinal study conducted in patients treated for PD-related peritonitis revealed elevation of serum leptin levels during acute peritonitis. The rise was contributed to anorexia in the earlier stage. In contrast, the serum adiponectin levels fell showing an inverse correlation between these two adipokines during acute peritonitis. Furthermore, the protracted course of inflammation even after bacterial cure of peritonitis was likely to cause the loss of lean body mass and to increase mortality [47].

## 5. Persistent Release of Pro-Inflammatory Mediators in Patients under Maintenance PD or after an Episode of Peritonitis

Patients on maintenance PD have increased intraperitoneal levels of hyaluronan and cytokines including IL-1*β*, IL-6, and TGF-*β* [48, 49]. Chronic inflammation remains an important cause of morbidity in patients with end-stage renal failure. The main causes for inflammation in CAPD are PD-related peritonitis and exit site infection [50]. Patients on PD with peritonitis may experience prolonged inflammation even when clinical evaluation suggests resolution of PD-related peritonitis [51]. The highly sensitive C-reactive protein (hs-CRP) remains significantly higher than baseline even by day 42 after an episode of peritonitis [47]. There was persistent release of Neutrophil Gelatinase-Associated Lipocalin (NGAL) in the peritoneal dialysate effluent (PDE) collected following an acute episode of CAPD-related peritonitis. NGAL synthesis is specifically induced in HPMC by IL-1*β* during peritonitis [52]. Interestingly, NGAL is also produced by adipocytes [53]. NGAL markedly affects the secretion of leptin and adiponectin by adipocytes, and acts as a negative regulator of inflammatory activity and inflammation-mediated adipocyte dysfunction. Incubation of HPMC with recombinant NGAL reverses the up-regulation of Snail and vimentin induced by TGF-*β*. Our data suggest that NGAL exerts a protective effect by modulating the epithelial-to-mesenchymal transition activated by peritonitis [52].

## 6. Role of Stem Cells from Adipose Tissue in Serosal Repair during CAPD

It has been shown that daily instillation of PDF for 5 weeks in rats leads to an increased number of omental mast cells and milky spots as well as damage to the mesothelial cell layer covering the peritoneum membrane [54]. Most interestingly, electron microscopy reveals that the severely damaged mesothelial cells are able to regenerate a good monolayer upon three months' rest of the peritoneum. The exact mechanism regulating this reversibility is not completely understood. Adipose tissues-derived SVF contains pluripotent mesenchymal stem cells that can regenerate damaged tissue [55]. An abundance of progenitor cells is also found in omentum [56]. Introduction of a foreign body into the peritoneal cavity further enhances the healing capability of the omentum by causing it to expand, surround the foreign body, and transform itself from mostly fatty tissue [56]. This transformed tissue (the activated omentum) contains abundant progenitor cells positive for CXCR-4 or Wilm's tumor-1 (WT-1), and is also rich in growth and angiogenic factors [56]. Activated omentum also facilitates liver regeneration following traumatic injury [57]. SVF cultured from omentum expresses pluripotent markers, produces high amounts of VEGF, and engrafts to injured sites [58]. These observations support a regenerative potential of mesothelium although the underlying mechanism remains undefined. The relative contribution of mesothelial cells, SVF or adipocytes in the adipose tissue and the relevant mechanism involved in the healing process of mesothelium after CAPD have not been well characterized. 

During peritoneal dialysis, the undesirable microenvironment, chronic inflammation, and previous peritonitis all impose stress, causing damage to the peritoneal membrane. Remesothelialization or healing is possible if the peritoneum is allowed to rest [54]. Regeneration or healing of the mesothelium does not occur solely by centripetal migration of cells from the wound edge. It has been proposed that pluripotent cells beneath the mesothelium migrate towards the surface and differentiate into mature mesothelial cells [79–81]. Others suggest that the new mesothelium originates from a free-floating mesothelial cell or progenitors in the serosal fluid [82]. Different origins of cells in the regenerating mesothelium have been proposed and these include subserosal mesenchymal precursors, bone marrow-derived precursors, free-floating macrophages, and free-floating mesothelial cells. The exact identity of this cell population responsible for mesothelial repair remains uncertain. 

**Figure 1:**
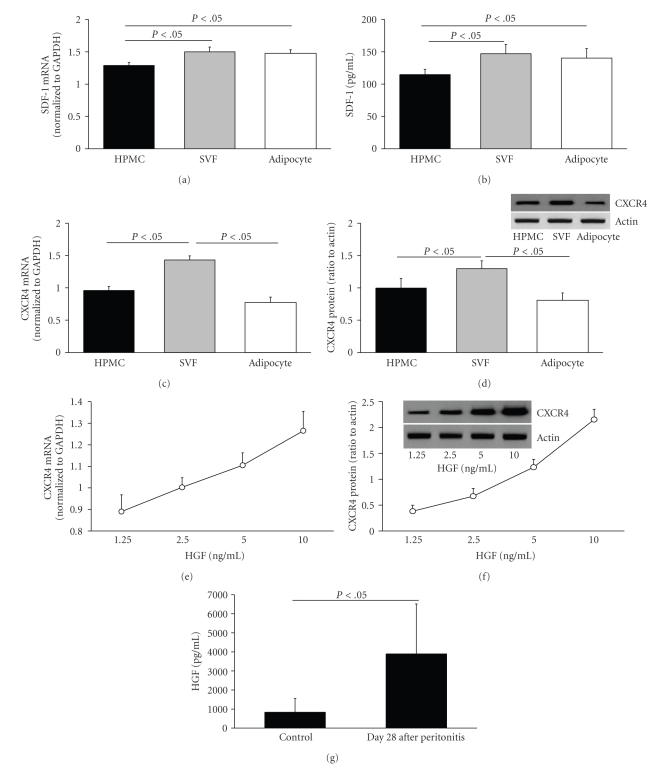
Constitutive expression of mRNA (expressed as amplicon ratio after normalized to GAPDH, measured by quantitative PCR), protein for G-protein-coupled chemokine receptor (CXCR4; expressed as ratio of densitometry data after normalized to GAPDH, measured by immunoblotting), and stromal derived factor-1 (SDF-1; measured by ELISA) in cultured human peritoneal mesothelial cells (HPMC), stromal vascular fraction (SVF), and adipocytes from human omental tissue (a to d). The CXCR4 expression in HPMC was up-regulated in a dose-dependent manner with hepatocyte growth factor (HGF) after 4 hours culture (e and f). Overnight PD effluent fluid (*n* = 15) was collected from CAPD patients on day 28 after the onset of peritonitis. Control PD effluent fluid (*n* = 15) was obtained in CAPD patients without previous history of peritonitis. The concentration of the HGF in PD effluent fluid was measured by ELISA. Persistent release of HGF in PD effluent was observed at day 28 after peritonitis in CAPD patients (g). These data are from our unpublished studies.

**Figure 2:**
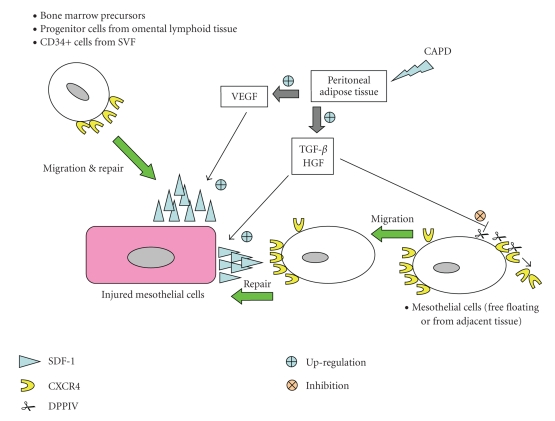
Schematic model illustrates the roles of adipokines or cytokines from adipose tissue on the repair of mesothelium under the context of CAPD. DPPIV indicates aminopeptidase dipeptidyl peptidase IV; CXCR4, G-protein-coupled chemokine receptor; SDF-1, stromal derived factor-1; SVF, stromal vascular fraction; TGF-*β*, transforming growth factor-*β*; TNF-*α*, tumor necrosis factor-*α*; VEGF, vascular endothelial growth factor.

Normal stem cells, mobilized from the bone marrow or resident in damaged tissue, play a pivotal role in tissue regeneration or healing after injury [83]. The *α*-chemokine stromal-derived factor-1 (SDF-1) and its unique G-protein-coupled chemokine receptor (CXCR4) constitute the SDF-1/CXCR4 axis that regulates the trafficking of stem cells during the repair of damaged tissues. SDF-1 is involved in the regulation of CXCR4+ progenitor cell trafficking [84–86]. Proper functioning of the SDF-1/CXCR4 axis plays a pivotal role in the healing and regenerative processes of damaged tissue [87], and this may be relevant to the repair of peritoneal membrane after CAPD. Accumulation of these progenitor cells in peritoneal tissues is affected by a cascade of inflammatory mediators produced by peritoneal cells (including macrophages, mesothelial cells, endothelial cells, and adipocytes) following long-term exposure to PDF during peritoneal dialysis. Unpublished data from our laboratory and from the literature [88, 89] demonstrate that SDF-1, CXCR4, as well as the endogenous aminopeptidase dipeptidyl peptidase IV (DPPIV or CD26 that controls the degradative pathway of the SDF-1) are expressed by HPMC (Figures 1(a) to 1(d), unpublished data). Notably, peritoneal permeability in CAPD patients with frequent peritonitis deteriorates with parallel increased expression of TGF-*β* in dialysate [90]. The SDF-1 expression is up-regulated in damaged tissue following TGF-*β* treatment leading to an increased migratory potential of CXCR4 bearing cells (including HPMC and progenitor cells from the bone marrow or adipose tissue) to the SDF-1-positive niche [89]. Other cytokines including HGF and VEGF may also participate in the up-regulation of SDF-1 synthesis in injured tissue. Up-regulation of the SDF-1 expression implicates the reepithelialization of denuded basement membrane at the site of peritoneal injury. This hypothesis is supported by the observation of a time- and dose-dependent reduction of DPPIV and E-cadherin expression in HPMC following TGF-*β*-induced morphological change. Following the inhibition of DPPIV, degradation of CXCR4 is retarded and hence significantly enhances the migratory potential of CXCR4 positive HPMC towards the SDF-1 gradient in the injured tissue. Apart from TGF-*β*, HGF also affects the SDF-1/CXCR4 axis. HGF increases the CXCR4 expression and SDF-1 production in glioma and facilitates their invasion [91, 92]. HGF released during peritonitis also alters mesothelial cell phenotype and function [93]. We observe a dose-dependent up-regulation of CXCR4 expression in HPMC by HGF (Figures 1(e) and 1(f), unpublished data). The dialysate level of HGF remains persistently elevated even 28 days after an episode of peritonitis (Figure 1(g)). The pleiotropic HGF may initially affect the mesothelial healing by promoting mesothelial cell growth, but can also contribute to peritoneal fibrosis by stimulating cell detachment with mesothelial denudation and collagen synthesis [93, 94]. The pathophysiological impact of prolonged release of these pro-inflammatory mediators on the SDF-1/CXCR4 axis and the mesothelial healing remains to be examined. Figure 2 is a schematic outlining the potential role of peritoneal adipokines and their interplay with the SDF-1/CXCR4 axis in regulating the regeneration process of the mesothelium in CAPD.

## 7. Conclusion

Long-term peritoneal dialysis is often associated with structural alterations of the peritoneal membrane that are closely related to chronic local as well as systemic inflammatory responses. It is evident that peritoneal mesothelial cells, fibroblasts, and macrophages exert their effects on peritoneal membrane during PD. Increasing evidences reveal that peritoneal adipose tissue also plays an important role in the structural and functional alterations during PD. In particular, adipocytes release secretory adipokines and cytokines that play modulating roles in the inflammatory cascade and healing response of the mesothelium in PD. In the present review, we summarized the relevance of adipose tissue associated adipokines and cytokines in PD, with focuses on recent data related to the leptin synthesis by peritoneal adipocytes and the associated cellular crosstalk with mesothelial cells. The possible involvement of the SDF-1/CXCR4 axis and adipose tissue-derived mediators in the regeneration process of the injured mesothelium after PD was also discussed. In order to better preserve the integrity of the peritoneal membrane, which facilitates long-term CAPD, novel studies designed to elucidate the detailed interaction between different peritoneal cellular components with the adipocytes in the context of PD should be undertaken. Further studies on the identity of peritoneal progenitor cells and the precise role of the SDF-1/CXCR4 axis in maintaining the peritoneal membrane function for peritoneal dialysis are warranted.

## Figures and Tables

**Table 1:** Major adipokines and cytokines released from adipose tissue.

Adipokine/cytokine	Cellular source in adipose tissue	Inflammatory effect	Relevance to PD	References
Leptin	Adipocytes	Pro-inflammatory	Serum and dialysate leptin increased after PD	[14, 59–61]
			Leptin augmented myofibroblastic conversion of HPMC	
Adiponectin	Adipocytes	Antiinflammatory	Glucose-based PDF increased plasma leptin/adiponectin	[62–64]
			Level in PD patients may indicate of cardiovascular disease risk	
Resistin	Macrophages	Pro-inflammatory	Level correlates with fat mass and triglycerides in PD patients	[64–66]
	Adipocytes			
Visfatin	Macrophages	Pro-inflammatory	Serum visfatin levels were higher in the PD patients	[67]
	Adipocytes			
RBP-4	Adipocytes	Pro-inflammatory	RBP-4 is significantly increased in end-stage renal disease	[68, 69]
NGAL	Mesothelial cells	Pro-inflammatory	Prolonged release of NGAL in dialysate following peritonitis	[52, 53]
	Adipocytes		NGAL was proposed as a novel early marker for acute renal failure	
TNF-*α*	Adipocytes	Pro-inflammatory	TNF-*α* production by macrophage was reduced by low pH and lactate in PDF	[60, 70, 71]
	Macrophages		Adipose-derived TNF-*α* inhibited leptin production	
	Mesothelial cells			
	Endothelial cells			
IL-6	Macrophages	Pro-inflammatory	Plasma and dialysate IL-6 were associated with high peritoneal solute transport rate	[72–74]
	Adipocytes		Mesothelial cells released IL-6 upon exposure to the spent dialysate or IL-1*β*	
	Mesothelial cells			
	Endothelial cells			
Apelin	Adipocytes	Pro-inflammatory	TNF up-regulated apelin expression in adipose tissue	[71, 75]
MCP-1	Macrophages	Pro-inflammatory	MCP-1 was up-regulated by TNF-*α* and regulated the differentiation of adipocytes	[76–78]
	Adipocytes			
	Preadipocytes			

## Abbreviations

AGE: Advanced glycation end products
DPPIV or CD26: Aminopeptidase dipeptidyl peptidase IV
BMI: Body mass index
CAPD: Continuous ambulatory peritoneal dialysis
GDP: Glucose degradation products
CXCR4: G-protein-coupled chemokine receptor
HGF: Hepatocyte growth factor
hs-CRP: Highly sensitive C-reactive protein
HPMC: Human peritoneal mesothelial cells
IGF-1: Insulin growth factor-1
IL: Interleukin
NGAL: Neutrophil Gelatinase-Associated Lipocalin
PDE: Peritoneal dialysate effluent
PD: Peritoneal dialysis
PDF: Peritoneal dialysis fluid
SDF-1: Stromal derived factor-1
SVF: Stromal vascular fraction
TGF-*β*: Transforming growth factor-*β*
TNF-*α*: Tumor necrosis factor-*α*
VEGF: Vascular endothelial growth factor
WT-1: Wilm's tumor-1
ZO-1: Zonula occludens protein 1.
